# Evaluation and Selection of Interspecific Lines of Groundnut (*Arachis hypogaea* L.) for Resistance to Leaf Spot Disease and for Yield Improvement

**DOI:** 10.3390/plants10050873

**Published:** 2021-04-26

**Authors:** Nicholas N. Denwar, Charles E. Simpson, James L. Starr, Terry A. Wheeler, Mark D. Burow

**Affiliations:** 1 Savanna Agricultural Research Institute, Tamale P.O. Box TL52, Ghana; nicholasdenwar@yahoo.com; 2 Department of Plant and Soil Science, Texas Tech University, Lubbock, TX 79409, USA; 3 Texas A&M AgriLife Research, Stephenville, TX 76401, USA; c-simpson@tamu.edu; 4 Department of Plant Pathology and Microbiology, College Station, Texas A&M University, Corpus Christi, TX 77843, USA; 47sail@gmail.com; 5 Texas A&M AgriLife Research, Lubbock, TX 79403, USA; ta-wheeler@tamu.edu

**Keywords:** synthetic amphidiploid, leaf spot resistance, cultivated peanut, wild species

## Abstract

Early and late leaf spot are two devastating diseases of peanut (*Arachis hypogaea* L.) worldwide. The development of a fertile, cross-compatible synthetic amphidiploid, TxAG-6 ([*A. batizocoi* × (*A. cardenasii* × *A. diogoi*)]^4x^), opened novel opportunities for the introgression of wild alleles for disease and pest resistance into commercial cultivars. Twenty-seven interspecific lines selected from prior evaluation of an advanced backcross population were evaluated for resistance to early and late leaf spot, and for yield in two locations in Ghana in 2006 and 2007. Several interspecific lines had early leaf spot scores significantly lower than the susceptible parent, indicating that resistance to leaf spot had been successfully introgressed and retained after three cycles of backcrossing. Time to appearance of early leaf spot symptoms was less in the introgression lines than in susceptible check cultivars, but the opposite was true for late leaf spot. Selected lines from families 43-08, 43-09, 50-04, and 60-02 had significantly reduced leaf spot scores, while lines from families 43-09, 44-10, and 63-06 had high pod yields. One line combined both resistance to leaf spot and high pod yield, and several other useful lines were also identified. Results suggest that it is possible to break linkage drag for low yield that accompanies resistance. However, results also suggest that resistance was diluted in many of the breeding lines, likely a result of the multigenic nature of resistance. Future QTL analysis may be useful to identify alleles for resistance and allow recombination and pyramiding of resistance alleles while reducing linkage drag.

## 1. Introduction

Early leaf spot (ELS) (caused by *Passalora arachidicola* (Hori) U Braun [*Cercospora arachidicola* S. Hori]) and late leaf spot (LLS) (caused by *Nothopassalora personata* (Berk. and M.A. Curtis) U. Braun, C. Nakash, Videira and Crous [*Cercosporidium personatum* [Berk. and Curtis] Deighton]) diseases are two of the most yield-limiting biotic stresses in peanut (*Arachis hypogaea* L.) production worldwide [1], causing yield losses of up to 50% [2,3] or even 70% in West Africa [4,5]. Although it has been suggested that resistance to each of these fungal diseases is inherited independently [6], both often occur together, with one predominating in different years and locations, making it imperative to deploy resistance to both pathogens. In the United States these diseases are controlled by fungicides, but application increases production costs by about 10% [7]. A study in Ghana [8] has confirmed that foliar application of fungicides can increase biomass and kernel yields in rainfed peanuts by 39% and 75%, respectively. However, the use of fungicides is not feasible for peasant farmers in Ghana and in most parts of West Africa. Credit facilities for the purchase of inputs and the input availability and delivery systems are not adequately developed. Various biological control agents have been proposed for control of leaf spots [9,10,11]; however, the limited effectiveness of these make them rarely suitable by themselves and they have been rarely used in practice. Better results have been obtained by combining varietal resistance with chemical or biological control and in some cases improved weed management [12,13]. In any case, effective control of these diseases for these farmers includes the use of host plant resistance.

Cultivated peanut [*Arachis hypogaea* L. (2*n* = 4x = 40)] is an allotetraploid that is native to South America, and contains two genomes that originated from different diploid wild species ancestors [14]. Earlier investigators [15,16,17] reported the existence of high levels of resistance in wild species in section *Arachis* to many pests and diseases. Attempts to utilize wild species sources have been difficult because introgressing wild alleles into *A. hypogaea* is complicated by genomic (A and B genomes) and ploidy (diploid and tetraploid) barriers [16].

Wild species introgression efforts have had some notable successes in peanut, namely in introduction of resistance to the root-knot nematode *Meloidogyne arenaria* (Neal) Chitwood. A cross was made between the A-genome diploid wild species *A. cardenasii* and the cultivated *A*. *hypogaea* [18,19], followed by doubling the triploid hybrid to the hexaploid level, followed by backcrossing to *A. hypogaea* and multiple generations of selfing to recover the tetraploid level. This cross resulted in introduction of alleles for resistance to nematodes and identification of markers to two linked genes associated with a reduction in egg counts or galling [20].

A second route for introduction of wild species alleles has also been employed, and which has resulted in the release of nematode-resistant cultivars. A three-way cross, originally proposed by Smartt et al. [21] to introduce alleles for leaf spot resistance, was made, resulting in development of a synthetic amphidiploid, TxAG-6 ([*A. batizocoi* × (*A. cardenasii* × *A. diogoi*)]^4x^) [17]. This was backcrossed to a component line of the runner cultivar Florunner [22], which was a highly successful cultivar grown for three decades, but lacking significant resistance to disease or pests. Six commercial nematode-resistant cultivars (COAN, NemaTam, Tifguard, Webb, TifN/V OL, and Georgia 14N) resulting from this cross have been released [23,24,25,26,27,28]. The latter five were developed using COAN as allele donor, and many of these cultivars were developed using DNA markers to the *Ma-1* gene [29,30,31,32]. However, this resistance was based on selection for a single chromosomal region which gave near-immunity to nematodes.

Tanksley and Nelson [33] proposed the advanced backcross-QTL method as an effective way to couple population advancement with gene discovery. Demonstrated benefits included making introgressed chromosome segments available in a suitable genetic background, sufficient polymorphism for mapping using the markers of that day, and well as reduced genetic background differences for enhancing the statistical power of QTL analysis, and potential to identify cryptic QTLs not expected based on the phenotypes of the parents. Using this approach in peanut, it was demonstrated that seven additional nematode resistance QTLs are present in a re-synthesized BC_3_ population and are spread over multiple chromosomes [34]. Most of these additional QTLs have weaker phenotypic effects than the *Ma-1* allele and were missed in the initial work, which relied on bulked segregant analysis. It is not known whether any of these have been incorporated into any of the commercial cultivars.

In general, introgressing useful alleles from wild species into cultivated crops has challenges because the beneficial alleles for yield and seed quality traits are often in tight linkages with undesirable traits that require several cycles of backcrossing to recover most of the desirable agronomic traits. In the case of the first nematode-resistant cultivar COAN, developed after five backcross generations, resistance was accompanied by linkage drag for low yield. It took two additional cycles of backcrossing to break this linkage, and the cultivar NemaTAM was released as a result [24].

In the case of leaf spots, it is generally considered that resistance is a multigenic trait, unlikely to be controlled by a single major gene [35,36,37], with many components, including initial infection, lesion size, sporulation and defoliation contributing to resistance [38,39,40,41]. High levels of resistance have also been associated with low yield suggesting linkage or pleitropic effects [42], thus breeding for high-yielding cultivars with resistance requires this linkage to be broken.

QTL analysis has in general confirmed these expectations. Khedikar et al., (2010) [43] reported in a cross between TAG24 and GPBD4 the identification of 11 QTLs for LLS that explained from 1.7% to 6.5% of phenotypic variation in three environments. Likewise, 11 QTLs with small-to-moderate effect on resistance to rust were identified, with 1.7% to 7.0% contribution to phenotypic variance each; however, one major QTL accounting for 6.9% to 55.2% of phenotypic variance (depending on environment) was identified, demonstrating that major-effect QTLs do exist for resistance to foliar disease. SNP chip analysis [44] has identified chromatin from *A. cardenasii* in GPBD 4 and leaf spot-resistant cultivars Bailey [45] and NkatieSARI [46]. Leal-Bertioli et al. [47] reported the mapping of 35 candidate genes and five quantitative trait loci (QTLs) for late leaf spot disease resistance and also indicated several regions within the *Arachis* genome involved in disease control.

Starting with TxAG-6, a new set of backcross materials was developed using the adapted cultivar Florunner [22] as the recurrent parent, and a genetic map was made from the BC_1_ generation [48]. BC_3_F_1_ single plants developed by the AB-QTL method were tested in the greenhouse for resistance to early leaf spots, and significant differences in lesion number and diameter, incubation period, latency period, and chlorosis were found. Burow et al. [49], using RFLP markers identified three QTLs for incubation period and one each for latency period, lesion number and diameter. QTLs for latency period and lesion number were overlapping, suggesting either linkage between the two or a QTL with pleiotropic effects. Selected progeny of these were advanced and were tested in the field for resistance to rust, early and late leaf spot in Texas, Burkina Faso and Ghana in a continuing effort to develop improved cultivars with acceptable agronomic characters and resistance to leaf spot diseases. Analyses of field data generated in Texas and West Africa over several years [50] was used to select backcross families with resistance to rust, early and late leaf spots, as well as having high yield and other agronomic characters.

However, there was the need to do an in-depth characterization of specific lines to identify those with adaptation to northern Ghana where over 90% of peanut production occurs in that country. Given the similarities in results between the U.S. (Texas), Ghana, and Burkina Faso in previous work [50], it is expected that results of characterization of theses breeding lines will be broadly applicable.

Our hypotheses are that (1) given the high levels of resistance to leaf spots in wild species, it is possible to introduce alleles for resistance to leaf spots into the cultivated species, and that (2) given the linkage drag for yield observed in COAN and the large number of QTLs for leaf spot resistance observed in previous QTL studies, we expect that multiple generations of backcrossing will be needed to break linkage drag for yield. We expect that it will be possible to identify some accessions with competitive yield and resistance to leaf spots.

The objectives of this study were to test these hypotheses by further field evaluation of selected BC_3_-derived interspecific lines of peanut for yield and resistance to early and late leaf spot diseases. According to our hypotheses, we expect to find some accessions with resistance to leaf spots, and competitive yield. Additionally, we expected to identify promising accessions that can be used as parents for donating useful alleles for varietal development.

## 2. Results

### 2.1. Leaf Spots

The combined analyses of early leaf spot scores of the 27 interspecific lines, three susceptible check varieties, and recurrent parent Florunner (Table 1) over environments in Ghana indicated that 20 entries had lower area under disease progress curves (AUDPC) than the susceptible local checks (Spanish, Chinese, and 55-437). Although there was a significant genotype × environment interaction for this and all other traits presented herein (Supplementary Table S1), the effect of genotype was nonetheless significant. In addition, in all Ghanaian environments where there was a statistically significant difference among accessions, the three Spanish check varieties Spanish, Chinese, and 55-437 were in the most susceptible statistical categories (denoted ‘a’ or ‘b’ in the means separation tests). Florunner (average score = 250) was less susceptible than Spanish (average score = 287) or 55-437 (average score = 267). Three introgression lines (50-02-04-01, 50-04-01-03, and 43-08-07-02) were also significantly less susceptible than Florunner across Ghanaian environments, and three additional introgression lines (43-09-03-02, 41-05-05-03, and 43-09-03-01) had means numerically lower than Florunner and were significantly lower in one or two environments, including Ghana and the U.S. Six introgression lines (50-02-04-01, 43-08-07-02, 41-05-05-03, 60-02-02-03, 43-09-03-02, and 19-05-06-03), including four of the aforementioned ones, also had means in the most resistant statistical category (for example, group ‘d’ at Manga in 2006) in three or four of the four environments where differences were significant. Materials were also tested in the United States at Yoakum, Texas. Four lines (43-08-07-02, 60-02-02-03, 43-09-03-02, and 41-05-05-03) were significantly more resistant than Florunner at Yoakum, as was 45-04-02-01, which had an overall mean higher than Florunner in Ghana, despite having a significantly lower early leaf spot score in two environments. In general, the most resistant entries in the Texas location were also the most resistant ones in Ghana.

For late leaf spot, there were no significant differences over environments in Ghana (Table 2). This was in part due to the lack of significant differences at Manga in 2007; however, 10 entries were statistically more resistant than the susceptible check varieties in two of the three remaining environments. The three entries (43-09-03-02, 60-02-02-03, and 43-08-07-02) that had the numerically lowest late leaf spot scores were also resistant to early leaf spot. One of these was among the three entries statistically better than Florunner for early leaf spot in Ghana, and all three were among the five entries with the lowest early leaf spot scores. Two entries (43-09-03-02 and 43-08-07-02) were in the most resistant statistical category in 3 environments, as was 19-05-06-03. At the Texas location, there were no differences between ratings in any of the entries (data not presented), as late leaf spot incidence was negligible.

### 2.2. Time to Appearance of Symptoms

Significant differences existed among the genotypes for time to appearance of the first early leaf spots. This ranged from 27.6 to 34.4 days after planting (DAP) (Table 3) at Nyankpala in 2006 to 36.6 to 48.9 DAP at Manga in 2007. Over all environments, there were large differences among introgression lines when symptoms first appeared. Many of the introgression lines developed initial symptoms earlier than the susceptible checks. Over environments, 19-05-06-03 and 70-03-07-01 were the earliest to manifest symptoms. Lines 45-04-02-01, and 50-04-01-03 were later than most of the introgression lines, but not different than the Chinese, Spanish, and 55-437 check varieties, which were numerically the latest three entries to manifest symptoms, and were the only accessions in the latest statistical category in two or three environments.

**Figure 1:**
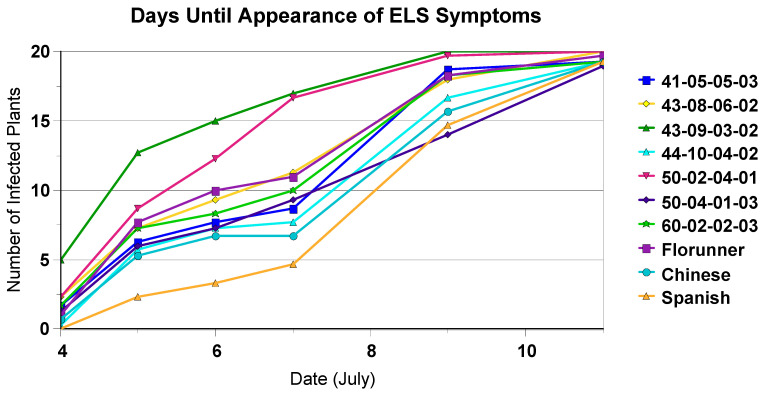
Days until appearance of early leaf spot symptoms, illustrating time to development of symptoms in 50% of plants at Nyankpala in 2006.

**Figure 2:**
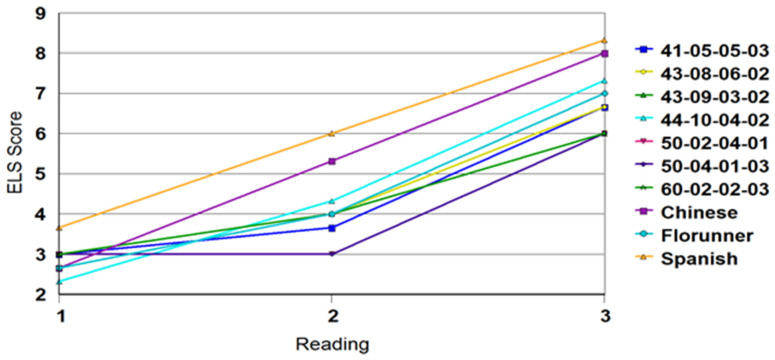
Disease ratings for early leaf spot using the Florida scale. Rating dates were after those used for the time course for development of symptoms, and were taken at 2-week intervals.

Comparing the time to development of early leaf spot symptoms to progression of early leaf spot suggested that the Spanish checks developed symptoms later than the resistant introgression lines (Table 3, Figure 1), but once disease had infected the plants, disease progressed faster in the susceptible materials (Figure 2). It should be noted that at least two of the early leaf spot ratings were taken at dates later than those used for scoring for the appearance of disease symptoms, depending on environment and timing of the onset of disease symptoms. In this case, by the time that the Florida scale ratings were taken, all the plants in a plot had disease symptoms.

For late leaf spot ratings, days to appearance of symptoms were taken in two environments (Manga 2006 and Nyankpala 2006) (Table 4), but results were different than for early leaf spot. Mean time for appearance of late leaf spots appeared for Spanish and Chinese earlier than for all introgression lines, 40 to 42 DAP at Manga, and 44 DAP at Nyankpala. Over environments, ten introgression lines had symptoms appear later than the susceptible checks and the recurrent parent Florunner, and seven lines were in the latest statistical category at both environments.

### 2.3. Pod Yield

Analysis of pod yields demonstrated that several interspecific lines outyielded the recurrent parent or susceptible checks. Examining means across environments, entries 43-09-03-01 and 44-10-01-02 outyielded the susceptible checks and recurrent parent Florunner; the mean of 63-06-08-02 outyielded susceptible checks across environments (Table 5). Lines 43-09-03-01 and 44-10-01-02 were in the top five yielders in all four environments, while 63-06-08-02 was in the top five in three environments, and 43-09-02-03, 43-08-06-02, and 43-09-03-02 were among the top five in two environments. Entries 46-06-07-02 and 50-02-04-01 were also in the highest-yielding statistical category in two environments.

By comparison, yields of the variety Spanish were numerically less than the overall mean in all four environments; 55–437 yielded less than the mean in three of three environments and Chinese yielded numerically less in one of two environments.

In 2006 in Manga, two introgression lines (43-09-03-01 and 44-10-01-02) yielded over 2 t/ha, while the susceptible checks all yielded 1.0 t/ha or less. Of the three highest-yielding entries in Manga 2006, two of them were of the family 43-09. In Nyankpala in 2006, the susceptible cultivar Chinese yielded numerically higher than all entries but not statistically greater than 43-08-06-02, 44-10-01-02, 41-10-01-03, 43-09-03-01, or 63-06-08-02. The shorter time to maturity of this variety compared to runners may be responsible for its higher relative yield. During the growing season in Nyankpala for 2006 there was cessation of rains in mid-October, less than three months into crop growth (rainfall data not shown). Rainfall post-planting was 400 mm, thus pod filling was very incomplete for late maturing entries, and yields were low even for early maturing entries such as Spanish and Chinese. The crop was in the field for 85 days, more than a month short of full maturity of approximately 120 days for most medium/late cultivars. Additionally, onset of leaf spot was early, and high disease ratings were obtained compared to other years and locations (Table 1 and Table 2).

In 2007, mean yields (kg/ha) were higher than in 2006. In Manga for 2007, line 43-09-03-01 was the highest yielding of all entries, with a value of 3.4 t/ha; 43-09-03-02 yielded well at 2.3 t/ha. A total of ten interspecific lines yielded over 2.0 t/ha at this location. Cultivar Florunner also yielded over 2.0 t/ha. Out of the 27 interspecific lines grown at Nyankpala in 2007, 21 had yields over one ton per hectare, 13 of them over 1.5 kg/ha, and six yielded over 2.0 t/ha.

### 2.4. Correlations

Correlation co-efficient values between the traits indicated a consistently strong positive association between AUDPC for early and late leaf spot, for both years and locations in Ghana. Correlation co-efficient values between early and late leaf spot ranged from 0.66 to 0.84 (Table 6). The second consistent result was negative correlations (r = −0.35, −0.31) between AUDPC for late leaf spot and time to appearance of late leaf spot symptoms at both locations in 2006.

In three environments (Nyankpala 2006, Manga 2007, and Nyankpala 2007), correlations between disease scores and yield were not significant statistically. In Manga 2006, there was a weak but significant positive correlation between ELS and LLS scores and yield. Correlation between ELS scores and time to appearance of ELS symptoms was nonsignificant in two environments, but positive at Nyankpala in 2006. This positive correlation indicates that the more resistant lines had earlier symptom expression. In this environment, there was a negative correlation between days to symptom expression and late leaf spot scores (more resistant started symptoms later). In 2007 at both sites, early and late leaf spot scores were correlated, but there was no relationship with day of initial symptom expression. Correlations excluded the susceptible checks, for which as noted earlier, time to appearance of symptoms was higher, but disease progression faster.

## 3. Discussion

### 3.1. Test of Hypotheses

Two hypotheses of this study were that (a) it is possible to introgress alleles for resistance to leaf spots into cultivated peanut from the wild species to improve resistance in the cultigen, and (b) that it would be possible to break linkage drag between resistance and low yield.

#### 3.1.1. Introgression

Results confirmed that resistance to early leaf spot had indeed been introgressed into the cultivated species, although results for late leaf spot were less clear. The study found numerous interspecific lines that were significantly more resistant to leaf spot than the standard susceptible check cultivars. However, the recurrent parent Florunner, which is considered to be highly susceptible in the U.S., was more resistant in Ghana than were the local susceptible check cultivars Spanish and Chinese, as well as the Senegalese variety 55-437. In comparing introgression lines to Florunner, three accessions had statistically lower early leaf spot ratings than Florunner in the pooled ANOVA across environments. Six accessions were more resistant than Florunner in two Ghanaian environments, and of these, three were more resistant also at Yoakum, TX. It should be noted that results from Nyankpala in 2007, where leaf spot pressure was lower, did not demonstrate the same pattern as the other three environments where leaf spot pressure was higher.

In the case of late leaf spot, there were no significant differences across all environments, although ten accessions were statically more resistant than Spanish, and five of these were numerically but not statistically superior to Florunner in two of four environments. Thus, if resistance to late leaf spot was present, it was at a degree too small to be distinguished statistically. Alternatively, late leaf spot resistance may have been present, but with measurements being confounded with early leaf spot measurements (see below).

Introgression of alleles from wild species was in accord with previous studies that demonstrated the presence of resistance to leaf spots in wild peanut species, including the parental species of TxAG-6. Indeed, the three parents of the TxAG-6 hybrid were selected based on reports of resistance to leaf spots, and the desire to introduce strong resistance into the cultivated species [15,17,19].

The quantitative nature of resistance to leaf spots is in contrast with that observed for resistance to the root-knot nematode, where one chromosomal locus with strong effect was responsible for a reduction of >90% in the number of nematode eggs per gram of root [29]. However, additional resistance alleles were identified later [30,32,34,51], and in QTL studies where it was possible to measure the strength of these alleles, many were shown to have smaller phenotypic effects than *Ma-1* [32,34].

The partial nature of resistance suggests that dilution of resistance may have occurred due to multiple backcrosses. Assuming that at least two [16,17,52], five [49,53], or 12 [43] genes for resistance to early or late leaf spot are present in the TxAG-6 parent, most resistance alleles would be absent in any given line. For a BC_3_F_1_ line, 25% of the resistance genes from the wild species amphidiploid would be expected to be present, and only in a heterozygous state. After multiple generations of selfing, one of eight genes would be expected to have resistance alleles present, and in the homozygous state. Depending on the number of resistance loci present in the original donor parent, this suggests from zero to two wild species resistance alleles present in any given introgression line, although more could be present depending on segregation of chromosomes and linkage among genes. In addition, if a large number (say from five to 12) of resistance genes were present in the TxAG-6 parent, it would be expected that the contribution of each would be relatively small, assuming that all made equal contributions to resistance.

#### 3.1.2. Breaking Linkage Drag

There was some support for the hypothesis of breaking linkage drag, at least in some lines. Evidence for breaking of linkage drag comes from identification of accessions with yields equal to or greater than Florunner, but with greater leaf spot resistance. Examples of this were 50-02-04-01, as well as 43-09-03-01 and 43-09-03-02.

However, data from other breeding lines suggest that linkage drag may still be present in the population. Evidence for this comes from the presence of only a few selections combining high yield and resistance. In many cases, the most resistant accessions generally were not the ones with the highest yield. Of the six introgression lines with lowest early leaf spot scores, four yielded numerically less than Florunner, and two numerically but not statistically higher. A second alternative is that there could be genetic linkage between disease resistance and late maturity, although this was not measured. The presence of nonsignificant correlations between leaf spot scores and yield in three of four environments might be evidence of breakage of linkage drag. However, under heavy leaf spot pressure, one might expect a negative correlation if resistance contributed to higher yield (see below for further discussion).

The results of this work stand in partial contrast to work performed previously [50], where larger differences among means of BC_3_-derived families were observed than those in the current experiment. In the former work, larger differences were observed among families for early and late leaf spot in Texas, and in Burkina Faso for overall leaf spot ratings (where early and late leaf spots were not distinguished). However, the smaller differences observed in the current work in Ghana are consistent with the smaller differences observed previously among families in Ghana. It is not known whether this could be due to differences in the pathogen among locations, or differences in environment.

### 3.2. Selections

A corollary of breaking linkage drag is that it is possible to identify breeding lines with superior resistance and yield that could be used as parents in a breeding program. This has been performed previously for nematode resistance and has resulted in the release of several nematode-resistant varieties. This implicit objective was fulfilled, identifying accessions with evidence of resistance to early leaf spot and with high yield; however, evidence for superior resistance to late leaf spot was equivocal.

First, for resistance, pooled data analysis across locations confirmed the superiority of several breeding lines for disease resistance. Given the large G × E interaction, identification of the best accessions was attempted using three methods (Supplementary Table S2). Up to six selections were made for each method. The first method was analysis of variance over the four pooled environments, with selections being statistically better than the checks. In this case, three introgression lines (50-02-04-01, 50-04-01-03, and 43-08-07-02) were more resistant than the susceptible checks and the recurrent parent Florunner (Table 1); accessions 60-02-02-03, 43-09-03-02, and 41-05-05-03 rounded out the top six based on means over Ghana environments. The second comparison was the number of environments in which an accession performed better than the susceptible checks and Florunner. Of the introgression lines with early leaf spot means over environments numerically lower than Florunner, four (50-04-01-03, 43-09-03-02, 41-05-05-03, and 43-09-03-01) were statistically lower than the checks and recurrent parent in at least two locations in Ghana, and four (43-08-07-02, 60-02-02-03, 43-09-03-02, and 41-05-05-03) were lower in at least one location in Ghana plus at Yoakum. The third comparison counted the number of Ghanaian environments in which each accession was in the most resistant statistical group; here 43-09-03-02, 41-05-05-03 and 60-02-02-03 were counted 4 times, and 19-05-06-03, 50-02-04-01, and 43-08-07-02 were counted three times each. Counting only the accessions that were in the top six selections using all three methods, three lines were selected–these were 43-08-07-02, 60-02-02-03, and 43-09-03-02; 43-08-07-02 was the only selection in the top three for all three methods.

For late leaf spot (Table 2), there were no significant differences over all environments; however, five introgression lines (60-02-02-03, 43-09-03-02, 43-08-07-02, 50-04-01-03, and 43-09-03-01) had the lowest numerical means. Entry 60-02-02-03 had the lowest mean numerically. Ten interspecific lines were superior to susceptible checks at two environments (43-09-03-02, 43-08-07-02, 50-04-01-03, 43-09-03-01, 19-05-06-03, 41-05-05-03, 44-10-04-02, 46-06-04-01, 41-10-03-03, and 45-04-02-01). Only four lines (43-09-03-02, 50-04-01-03, 43-09-03-01, and 45-04-02-01) were statistically superior to Florunner at even one environment. Three lines (43-09-03-02, 43-08-07-02, and 19-05-06-03) were in the most resistant statistical group in all three Ghanaian environments, and 13 additional lines were in the most resistant statistical group in two environments. Four breeding lines were common to all three selection methods (Supplementary Table S2), namely 43-09-03-02, 43-08-07-02, 50-04-01-03, and 43-09-03-01; 60-02-02-03 could be selected based on commonality among two selection methods.

For late leaf spot, that there were no statistically significant differences in mean over environments has several potential explanations. One is that the donor parent TxAG-6 may be less resistant to late leaf spot than early leaf spot. Another possibility is that there may be more and weaker alleles for resistance to late leaf spot than to early leaf spot. A third possible explanation is that scoring of late leaf spot is confounded with scoring for early leaf spot, and therefore it is more difficult to identify differences for late leaf spot.

For yield, two interspecific lines (43-09-03-01 and 44-10-01-02) were superior to Florunner in the pooled ANOVA across locations (Table 5). One additional line (63-06-08-02) was statistically higher yielding than Spanish. These three introgression lines outyielded the susceptible recurrent parent Florunner by 36% or more. Three additional lines (43-09-02-03, 43-08-06-02, and 43-09-03-02) filled out the top six. Four lines (43-09-03-01, 44-10-01-02 plus 43-09-02-03 and 43-09-03-02) had means numerically higher than both Florunner and Spanish in all four environments, and these plus 63-06-08-02, 46-06-07-02, 19-05-06-03, and 50-02-04-01 had means higher in three of four environments. Two lines (43-09-03-01 and 44-10-01-02) were in the top statistical yield category over all four environments in Ghana; 63-06-08-02 was in the top statistical yield category in three environments; and four lines (43-09-02-03, 43-08-06-02, 43-09-03-02, 46-06-07-02) were in the top statistical yield category in two environments. When selections were made according to selections made using all three methods, 43-09-03-01, 43-09-03-02, 44-10-01-02, and 63-06-08-02 were represented in all three listings.

Surprisingly, only three introgression lines significantly outyielded the susceptible checks Spanish (2006 and 2007), and only four outyielded Chinese (Manga, 2006). One explanation is that Chinese is adapted to the area and is widely grown. Additionally, Chinese and Spanish have Spanish growth habits but the introgression lines are runners, and the Spanish type is typically earlier in maturity by several weeks compared to runners. The short growing season in northern Ghana would favor early-maturing accessions, as terminal drought ends the growing season. This is consistent with yield data from Nyankpala in 2006, in which Chinese yielded the best in the short 85 day growing season that year.

For making selections for both resistance to leaf spots and for high yield (Supplementary Table S2), few lines possessed both high levels of disease resistance and high yield. Selecting based on mean ranks summed across ELS, LLS, and yield, line 43-09-03-02 was the top breeding line, and 43-09-03-01, 43-08-07-02, 50-02-04-01, 19-05-06-03, and 43-08-06-02 made the top six. Based on number of environments where means were superior to susceptible checks, 43-09-03-02 was the top entry, with 43-09-03-01, 50-04-01-03, 41-05-05-03, 43-08-07-02, and 19-05-06-03 filling out the top six. Based on number of times in the most favorable statistical category, entry 43-09-03-02 had the highest score, followed by 19-05-06-03, 41-05-05-03, 50-02-04-01, and 43-09-03-01. Four lines were selections in all three methods of selection—43-09-03-02 was the top entry according to all three methods, and 19-05-06-03, 43-08-07-02, 43-09-03-01 were the other three selections. Three of these were derived from the same BC_1_ line (#43), and so might be expected to possess some alleles in common. Entries 41-05-05-03, 50-02-04-01, and 60-02-02-03 were selections common to two of the three methods, but 41-05-05-03 and 60-02-02-03 had pooled mean yields that were numerically lower than Florunner. These might be useful sources of resistance that might differ in alleles from the selections based on BC1-43, but their use may require breakage of linkage drag to low yield in additional generations of crossing and selection.

One of these accessions (43-09-03-02) is currently being used in marker-assisted backcrossing breeding programs in Texas and Ghana. In a similar evaluation of interspecific lines, Ouedraogo et al. [54] suggested that at least one interspecific line combined genes conditioning resistance to both leaf spot diseases and high yield in peanut, supporting our conclusion that TxAG-6 was an effective bridge in introgressing resistance alleles from wild ancestors of *Arachis* into cultivated peanut as the introgressed lines performed better than the recurrent parent in most of the traits studied.

### 3.3. Early and Late Leaf Spot Ratings

Distinguishing early from late leaf spot was possible, but may not have been reflected fully in the ratings. Measurements of early and late leaf spots were made in an effort to distinguish the two diseases. The two diseases can be distinguished from each other easily by physical appearance of the spots—there is a yellow halo around the brown early leaf spot lesions, and absence of the halo and black color for late leaf spot lesions. As there was a high number of spots of both diseases, scores at these infection levels were based primarily on defoliation, thus rating plants in the field independently for these two traits is difficult and sometimes misleading unless one disease is more prominent. In Yoakum, late leaf spot pressure was low, and so it was possible to make ratings for early leaf spot independently. In general, early leaf spot appears 2 to 4 weeks before late leaf spot, and thus rating for early leaf spot would be less prone to interference from late leaf spot than the opposite case. Given the high incidence of both diseases in Ghana, an alternative for distinguishing the two diseases would be by inoculated growth chamber or greenhouse studies with artificial inoculum.

Significant genotype × environment interactions were observed for all five traits measured. Differences in environment in Ghana involved rainfall between locations. Nyankpala is located in the Guinea Savannah zone and has a long-term average rainfall of approximately 1300 mm. Manga, on the other hand, is located in the Sudan Savannah, and has a lower annual rainfall of approximately 900 mm. An example of the differences could be seen at Nyankpala in 2006, where disease appeared early and high disease scores were obtained, but early cessation of rains resulted in poor yields. At Manga in 2007, onset of early leaf spot was later than at the other two environments where data were taken. Leaf spot ratings among the three environments with high leaf spot pressure were relatively consistent, but rankings at Nyankpala in 2007 were often different from the high-pressure environments. It is possible that this is associated with differences in the speed of development of symptoms.

Despite the presence of significant genotype × environment interactions, differences among genotypes were significant for all traits presented except for late leaf spot AUDPC. A comparison of the number of environments in which an accession was in the most favorable statistical category gave additional support to distinguishing accessions as resistant using means across locations. In addition, the classification of several accessions as resistant both in Ghana and in the U.S. also supported the hypothesis that there were genetic differences for resistance that were consistent across locations.

Additionally, given the wide differences in geography, it is possible that there are differences among the leaf spot pathogens. However, there are no published race-specific differentials for early leaf spot or late leaf spot pathogens. We cannot discount that pathogen differences within a species may exist, but even in this case, the similarity in rankings of the most resistant accessions in Ghana and Texas suggest a common genetic component of resistance.

An exception to this commonality of results between countries appears in comparison of ratings of Florunner in Ghana, which appeared to show that it had some degree of resistance to leaf spots in comparison to the susceptible Spanish checks. However, results from Yoakum in 2005, as well as other environments [55], demonstrate that Florunner is highly susceptible to leaf spots in Texas and numerous introgression lines are significantly more resistant than the recurrent parent. Unfortunately, we did not have seed of the Spanish checks available to plant at Yoakum in 2005, so it is not possible to compare the performance of Florunner to the susceptible Spanish checks at this location. One possibility is that there are differences among strains of the leaf spot pathogens between Texas and West Africa, but no study of this has been performed to date. Alternatively, this could be some effect of environment, or an interaction between infection and senescence of the earlier-maturing Spanish materials.

### 3.4. Time to Appearance of Symptoms and Disease Incidence

Unexpectedly, almost all the introgression lines had significantly shorter times to appearance of early leaf spot than did the susceptible checks. This apparent contradiction may be an effect of genetic background, in which the Florunner background of all introgression lines develop symptoms sooner than the Spanish background of susceptible check varieties. However, once symptoms developed on the susceptible Spanish varieties, disease progression was faster for early leaf spot. For late leaf spot, the susceptible Spanish checks developed symptoms earlier than the introgression lines. This may indicate that these materials were already succumbing to early leaf spot pressure, and were not able to fight off late leaf spot infection well.

Among the introgression lines, for early leaf spot, there was generally no correlation between AUDPC and time to appearance of symptoms. For late leaf spot, by contrast, there was a strong negative correlation between disease severity and time to appearance of symptoms. This may again signify some difference in response of the groundnut accessions to the two pathogens.

Early leaf spot symptoms appeared earlier at Nyankpala in both years than at Manga. The timing of the appearance of early leaf spot demonstrates differences due to environment. This was also observed by Pande et al. [55]. As the rainfall season normally starts earlier in Nyankpala than Manga, and total rainfall is also higher, the optimum humidity and temperature required to initiate infection is expected to occur earlier at Nyankpala. The rains started in Nyankpala in February of 2006 but it was not until April that rainfall was experienced in Manga; again, while there was rain in Nyankpala in March of 2007, no rain fell in Manga until April.

In field and greenhouse experiments, Pande et al. [55] observed significant differences among interspecific lines and between locations but not between years. They found that the range of incubation period was 8 to 15 days in the greenhouse but 10 to 17 days for the field study. In the current study, incubation period could not be obtained, since disease was initiated naturally. Field results, as reported by Ouedraogo [49], indicated the period to symptom initiation for early leaf spot to be in the range of 15 to 20 days for Yoakum, TX, comparable to what is reported by Tuggle [56] in the range of 18 to 21 days. In the current experiment, disease symptoms appeared later than this, indicating either longer incubation periods, or a delay between planting and presence of sufficient inoculum or interaction phenotype for conditions favorable for disease.

### 3.5. Correlations

Correlations between early and late leaf spot ratings were high, as has been observed previously. Correlations coefficients between early and late leaf spot AUDPC ratings ranged from 0.65 to 0.89, comparable to those obtained by Anderson et al. [37] that ranged from 0.41 to 0.86. They suggested the high correlation could be due to possible genetic linkage of genes for resistance, or host plant physiology that conferred resistance to both diseases in a population, or confounding of scoring for both diseases.

In the current study, there was a lack of correlation between early leaf spot score and time to appearance of early leaf spot symptoms among introgression lines; however, a negative correlation was found for these under late leaf spot. Chiteka et al. [57,58] found no correlation between resistance and time to appearance of symptoms; however, Dwivedi et al. [59] found that incubation period was negatively correlated with leaf spot score.

### 3.6. Utility of the AB-QTL Method

Tanksley and Nelson [33] proposed the AB-QTL method as a modification of the inbred-backcross method, to couple introgression with gene discovery. Advantages of this approach were putting introgressed chromatin into a suitable genetic background, breaking of linkage drag, and enhanced statistical power. It was also shown [60] that this could identify cryptic alleles present in the wild species, and two such QTLs for enhanced yield were discovered in *Oryza rufipogon*. By a similar procedure, additional QTLs for root-knot nematode resistance were found, and cryptic alleles were also found [34].

In the current work, the presence of several accessions with yield greater than the susceptible checks suggests either a favorable combination of alleles for yield and resistance to leaf spots, or perhaps the presence of cryptic alleles for yield. Molecular marker dissection of the response will be necessary to distinguish these.

However, the AB-QTL method has its limitations as is evident here. The current data from Ghana suggest that the introgression lines studied possess moderate resistance to leaf spots. Specifically, multiple cycles of backcrossing have apparently diluted the strength of resistance from what was expected based on reports of resistance of the wild species parents. We might expect few if any major QTLs, although the limited results from Yoakum presented herein may indicate a difference among locations for this. Nevertheless, the use of multiple backcrosses was necessary, as visual observation of BC_1_ and BC_2_ breeding lines (Simpson, Starr, and Burow, unpublished observation) in the greenhouse indicated that the BC_3_ generation from the TxAG-6 x Florunner cross was the first to generate significant numbers of progeny with agronomic characteristics similar to the cultivated recurrent parent.

### 3.7. Continuing Work

The identification of interspecific introgression lines with enhanced resistance to leaf spots offers the possibility of introduction of novel alleles for resistance into the cultivated species. To do this will involve use of selected breeding lines and crossing by elite materials lacking only resistance to leaf spots. Several introgression lines, including 43-09-03-02 are promising, as they offer both demonstrable resistance to early leaf spot, possibly resistance to late leaf spot, and yield as good as or superior to Florunner under disease conditions.

Development of SNP-based markers and QTL analysis would be beneficial in identification of QTLs. Previously, RFLP and SSR-based maps were developed for the earlier BC_1_ population [48]. RFLP markers for greenhouse response to early leaf spot have been identified [49], and SSR markers [61] for oil content and composition were identified in BC_3_F_1_ and BC_3_F_6_ materials, respectively. Use of SNP-based markers would be a substantial improvement in methodology, and allow high-throughput marker-assisted breeding. Initial efforts in this have been reported [62,63].

Carrying these alleles forward can be performed in two ways. The first is directly by marker-assisted backcrossing. The breeding line 43-09-03-02 when under disease pressure has resistance to early leaf spot significant enough to be detected, and has high enough yield to be useful without significant linkage drag. Selection of potential SNP-based QTLs would allow for selection of resistance QTLs in a backcross scheme.

A second, longer-term project would be selection for smaller introgressed chromosome segments due to genetic recombination, and intercrossing to recombine QTLs. Genetic mapping and QTL analysis are expected to identify breeding lines with relatively small introgressed chromosome segments less likely to be accompanied by linkage drag, and possessing genes conferring resistance to leaf spots. Hybridization of introgression lines possessing different, small chromosome segments containing QTLs for resistance has the potential to enhance resistance to levels similar in the wild species parental accessions, but without the undesirable alleles present in the wild species.

## 4. Materials and Methods

### 4.1. Plant Materials

The 27 interspecific lines were derived from interspecific crosses made between the amphidiploid TxAG-6 and the component line UF439-16-10-3-2 of the cultivar Florunner [22] and selected on the basis of previous analyses [17,34,48,49,50]. Designations of the introgression lines consist of four sets of two-digit numbers in the pedigree of the materials, referring to BC_1_ line, BC_2_ line, BC_3_F_3_ line, and BC_3_F_3_ single plant, respectively. Backcross materials used in Ghana in 2006 and 2007 were in the BC_3_F_6_ and BC_3_F_7_ generations, respectively, and in the BC_3_F_5_ generation in Texas in 2005. Cultivar Chinese (also known as Shi Tao Chi) was used as a susceptible check and is an early maturing, erect cultivar that has been grown by farmers for many decades in northern Ghana [64]. Spanish has similar characteristics as Chinese. Both have good confectionery and culinary qualities, but are highly susceptible to both diseases. The variety 55-437 is a drought-tolerant, disease-susceptible Spanish type peanut, commonly grown in Senegal [65].

### 4.2. Field Plot Design and Operations

All 27 interspecific lines were planted in all 5 environments, namely at Nyankpala, Northern Region (9°25′ N, 0°58′ W) and Manga, Upper East Region (11°01′ N, 0°16′ W), Ghana in 2006 and 2007 (BC_3_F_7_), and at the former Texas Agricultural Experiment Station experimental farm at Yoakum TX (29°16′ N, 97°07′ W) (BC_3_F_5_) in 2005. The design in all three years was a randomized complete block with three replications. Each plot in Ghana consisted of 2 rows at a planting distance of 60 cm between rows and 30 cm between plants, plots 5 m in length. Fertilizer was applied at the rate of 30 kg K_2_O and 60 kg P_2_O_5_ per hectare. Weeds were controlled by hand hoeing at 2 and 6 weeks after planting. Additional weed control was performed as necessary. Plants in Texas were grown in two-row plots 2.4 m in length, 1.5 m between ranges, and 0.91 m between plots. Spreader rows of the susceptible cultivar Florunner were planted between each pair of plots, so that each plot was adjacent to a spreader row. In addition, Florunner was planted at the front and rear of the test.

### 4.3. Defoliation Ratings

The severity of early and late leaf spot was estimated using the Florida scale (1–10) as follows: 1 = no leaf spot; 2 = very few lesions on leaves, none on the upper canopy; 3 = few lesions on the leaves, very few on the upper canopy; 4 = some lesions, with more on the upper canopy, 5% defoliation; 5 = lesions noticeable even on upper canopy, 20% defoliation; 6 = lesions numerous and very evident on upper canopy, 50% defoliation; 7 = lesions numerous on upper canopy, 75% defoliation; 8 = upper canopy covered with lesions, 90% defoliation; 9 = very few leaves remaining and those covered with lesions, 98% defoliation; and 10 = plants completely defoliated and killed by leaf spot [53].

Early leaf spot scores in Ghana were taken every two weeks beginning one month after emergence. Two ratings were taken at Manga in 2006, three ratings were taken at Nyankpala in 2006, and three ratings each were taken at Manga and Nyankpala in 2007. Late leaf spot scores were taken beginning two months after emergence, the same number of ratings as for early leaf spot. In Yoakum in 2005, only one rating was taken due to the distance between the site and the home base. Even though both early and late leaf lesions occurred on the same plant, they were distinguishable. Early leaf spot is characterized by a light brown lesion with a yellow halo as opposed to the dark brown or black lesions of late leaf spot that lack halos.

Data from Ghana were transformed using the area under the disease progress curve calculation to give a time-weighted mean of Florida scale ratings. This was needed because high disease pressure resulted in high defoliation in some environ-ments by the time of the last rating, making it difficult to distinguish differences among accessions. AUDPC gives a summary of the disease progress from the time of first rating to the last rating [66]:

(1) $$AUDPC=\sum_{i}^{n-1}\left(\frac{y_{i}+y_{i+1}}{2}\right)(t_{i+1}-t_{i})$$

where *y* = disease intensity, *n* = number of assessment times and *t* = time (in days) of the *i*th rating.

### 4.4. Time to Appearance of Symptoms

After emergence, each leaf of every plant for each entry was inspected periodically for the appearance of the first leaf lesion; intervals between ratings ranged from 1 to 7 days. The number of days from emergence to the appearance of the first lesion was recorded for that plant. Time to appearance of symptoms for the plot for each foliar disease was calculated by interpolation as the time at which 50% of the plants had at least one lesion from that disease. Ratings were taken at six dates in 2006 at Manga for late leaf spot, and six ratings were taken for early leaf spot and four for late leaf spot in 2006 at Nyankpala. In 2007, six early leaf spot ratings were taken at Manga, and five at Nyankpala. Due to distance and labor constraints, it was not possible to collect data on symptoms initiation period for early leaf spot at Manga in 2006, or for late leaf spot at Manga or Nyankpala in 2007.

Linear interpolation between time points to estimate the time (days after planting) at which 50% of the plants in a plot demonstrated symptoms was performed using the software program CalcIP6 (Supplementary Table S3).

### 4.5. Pod Yield

Yield per hectare was estimated by converting the pod yield per plot to kilograms per hectare by multiplying the pod yield per plot times the ratio of the area of a hectare divided by the area per plot.

### 4.6. Statistical Analysis

For analysis over environments, a mixed model was used, with environment and replication within environments as random effects, and accessions as fixed effects. Environments (year and location) were used instead of year and location separately to avoid imbalance in cases where data were not taken in one environment. Mean separation tests were performed using Fisher’s protected LSD when the analysis of variance indicated significant treatment effects at *p* ≤ 0.05 [67]. Data were analyzed using PROC GLM of SYSTAT 7.0 (SYSTAT Inc., Chicago, IL, USA). Pearson correlations were performed using PROC CORR of SYSTAT 7.0, for backcross introgression lines.

## 5. Conclusions

Results supported the hypothesis that it is possible to introgress alleles for resistance to early leaf spot from wild species into the cultigen by use of the synthetic amphidiploid TxAG-6 as a parent. Specific resistant accessions were identified. There is evidence that alleles for resistance to late leaf spot may have been introduced also, but evidence was not conclusive. Three accessions were identified with yields superior to the susceptible recurrent parent under disease conditions, suggesting a breakage of linkage between resistance and low yield, or at least that resistance in these materials was sufficient to overcome loss of yield in the recurrent parent under disease.

Lines with low early leaf spot and high yield were identified in the introgression population; these have potential use as parents for future population development. Resistance in introgression lines was lower than expected in Ghana, suggesting that repeated backcrossing resulted in a dilution of resistance alleles effective in that environment. Limited data from Texas suggested that resistant lines were more effective in that environment compared to the recurrent parent.

Identification of QTLs is expected to assist in use of these materials for marker-assisted backcrossing to develop leaf spot-resistant cultivars. Breakage of long introgressed chromosome regions and recombination of alleles by hybridization of different introgression lines is expected to make it possible to recover higher levels of resistance more characteristic of the wild species parents.

## Figures and Tables

**Table 1:** Response of cultivars to early leaf spot at five environments. Values are AUDPC scores for Manga and Nyankpala in Ghana, and Florida scale score for Yoakum, TX in the U.S.

Accession	Mean ^1^		2006 Manga		2006 Nyankpala		2007 Manga		2007 Nyankpala		2005 Yoakum		N MFG ^2^
Spanish	287	a ^3^	225	a	360	a	400	ns ^4^	163	a-d	n.d. ^5^		0
Chinese	n.d.		225	a	320	b	n.d.		n.d.		n.d.		0
55-437	267	b	216	a–c	315	bc	370		167	ab	n.d.		0
26-10-06-03	260	bc	215	a–c	285	c–e	375		165	a–c	7.33	a–e	0
45-03-06-01	258	b–d	225	a	280	de	375		152	a–h	7.16	a–e	0
31-04-10-03	257	b–d	225	a	295	b–d	360		147	b–h	6.00	c–f	0
31-04-05-02	256	b–d	205	b–d	295	b–d	370		155	a–f	7.00	a–e	0
63-04-02-03	256	b–d	210	a–d	295	b–d	375		142	c–h	7.66	a–d	0
63-04-02-02	256	b–d	210	a–d	275	d–f	380		157	a–f	6.66	a–f	0
50-04-03-03	253	b–d	215	a–c	255	e–g	380		163	a–d	8.33	ab	0
45-04-02-01	252	c–e	225	a	280	de	375		128	h	4.83	fg	2
44-10-04-02	252	c–e	200	cd	275	d–f	360		173	a	7.50	a–d	1
70-03-07-01	252	c–e	225	a	265	d–f	380		137	e–h	5.66	d–g	2
41-10-03-03	250	c–f	200	cd	280	de	355		167	a–b	7.33	a–e	1
63-06-08-02	250	c–f	220	ab	270	d–f	350		160	a–e	8.33	ab	0
Florunner	250	c–f	215	a–c	265	d–f	355		164	a–c	7.67	a–d	0
46-06-04-01	250	c-f	210	a–d	260	ef	375		153	a–g	8.17	ab	1
46-06-07-02	249	c–g	220	ab	265	d–f	370		142	c–h	8.17	ab	1
41-10-01-03	249	c–h	215	a–c	265	d–f	360		155	a–f	8.00	a–c	0
43-09-02-03	248	c–h	225	a	270	d–f	350		145	b–h	5.83	d–f	1
63-04-05-02	247	c–h	220	ab	270	d–f	350		149	a–h	7.33	a–e	1
43-09-03-01	246	c–h	220	ab	260	ef	375		128	h	5.83	d–f	1
44-10-01-02	245	d–h	215	a–c	275	d–f	350		141	c–h	5.67	d–g	1
19-05-06-03	245	d–h	204	b–d	255	e–g	375		146	b–h	8.00	a–c	3
43-08-06-02	245	d–h	210	a–d	265	d–f	365		139	d–h	8.50	a	1
41-05-05-03	244	d–h	195	d	255	e–g	375		150	a–h	4.91	fg	4
43-09-03-02	239	e–i	210	a–d	255	e–g	360		130	gh	5.33	e–g	4
60-02-02-03	236	f–i	205	b–d	255	e–g	335		150	a–h	4.67	fg	4
43-08-07-02	235	g–i	220	ab	245	fg	335		139	d–h	3.67	g	3
50-04-01-03	235	hi	220	ab	225	g	360		134	f–h	8.67	a	2
50-02-04-01	228	i	210	a–d	225	g	330		147	b–h	6.33	b–f	3
*p*	0.0044		0.0070		0.0001		0.2056		0.0207		0.0001		
Mean	250		215		273		364		150		6.82		
LSD	15		17		32				24		2.02		
CV	7.6%		4.7%		7.2%		6.5%		9.2%		15.1%		

^1^ Mean refers to a combined analysis for the four Ghana locations, but does not include the Texas location. ^2^ N MFG, number of environments in which the accession occurred in the most favorable [resistant] statistical group. ^3^ Values sharing the same letter are not statistically different at the *p* = 0.05 level of probability. ^4^ ns, not statistically significant. ^5^ n.d., no data.

**Table 2:** Response of cultivars to late leaf spot. Values are AUDPC scores.

Accession	Mean		2006 Manga		2006 Nyankpala		2007 Manga		2007 Nyankpala		N MFG ^1^
Spanish	238	ns ^2^	195	a ^3^	380	a	260	ns	118	a–d	0
Chinese	n.d. ^4^		195	a	340	b	n.d.		n.d.		0
55-437	227		185	a–c	345	b	250		129	ab	0
45-04-02-01	213		195	a	290	c–e	275		92	hi	0
26-10-06-03	210		185	a–c	280	c–g	255		121	a–d	0
31-04-10-03	210		190	ab	280	c–g	255		114	a–f	0
43-09-02-03	209		195	a	290	c–e	240		110	b–h	0
45-03-06-01	207		185	a–c	275	c–h	260		109	b–i	2
41-10-01-03	207		175	b–e	290	c–e	250		113	a–g	1
70-03-07-01	207		185	a–c	260	e–h	270		113	a–g	1
63-06-08-02	206		185	a–c	285	c–f	235		120	a–d	1
31-04-05-02	206		170	c–e	305	c	235		115	a–f	0
41-10-03-03	205		165	de	270	d–h	255		131	a	2
44-10-01-02	204		180	a–d	295	cd	235		107	c–i	1
63-04-02-02	204		170	c–e	270	d–h	260		114	a–f	2
63-04-02-03	203		175	b–e	290	c–e	240		109	c–i	2
46-06-07-02	203		185	a–c	260	e–h	255		114	a–f	1
63-04-05-02	202		180	a–d	280	c–g	240		107	c–i	1
46-06-04-01	202		165	de	265	d–h	255		122	a–d	2
44-10-04-02	201		165	de	280	c–g	235		125	a–c	1
43-08-06-02	201		180	a–d	275	c–h	245		103	d–i	2
50-02-04-01	200		180	a–d	260	e–h	255		104	d–i	2
50-04-03-03	199		175	b–e	250	gh	260		112	a–h	2
41-05-05-03	198		160	e	265	d–h	250		119	a–d	2
19-05-06-03	197		167	de	265	d–h	250		107	c–i	3
43-09-03-01	196		185	a–c	255	f–h	250		96	f–i	2
50-04-01-03	195		190	ab	245	h	250		93	g–i	2
Florunner	193		170	c–e	260	e–h	225		118	a–d	2
43-08-07-02	193		175	b–e	255	f–h	245		97	e–i	3
43-09-03-02	191		175	b–e	260	e–h	240		89	i	3
60-02-02-03	190		170	c–e	250	gh	230		110	b–h	2
*p*	0.1024		0.0003		0.0001		0.7600		0.0106		
Mean	204		179		280		249		111		
LSD			17		33				21		
CV	12.1%		5.7%		7.2%		9.1%		10.4%		

^1^ N MFG, number of environments in which the accession occurred in the most favorable statistical group. ^2^ ns, not statistically significant. ^3^ Values sharing the same letter are not statistically different at the *p* = 0.05 level of probability. ^4^ n.d., no data.

**Table 3:** Number of days for initial appearance of symptoms of early leaf spot on 50% of plants.

Accession	Mean		Nyankapala 2006		Manga 2007		Nyankpala 2007		N MFG ^1^
55-437	36.30	a ^2^	34.42	a	47.17	ab	27.33	a-c	3
Spanish	36.07	ab	31.09	bc	47.37	ab	29.76	a	2
Chinese	n.d.^3^		30.29	b–g	n.d.		n.d.		1
50-04-01-03	34.79	a–d	30.43	b–g	44.10	bc	29.85	a	1
45-04-02-01	34.06	a–e	30.11	b–h	43.10	cd	28.96	a	1
50-04-03-03	33.87	b–f	30.91	b–e	42.63	c–e	28.07	a–c	1
43-09-03-02	33.71	c–f	27.64	l	44.03	bc	29.46	a	1
43-08-06-02	33.68	c–g	29.30	d–l	43.13	cd	28.62	a	1
43-08-07-02	33.60	c–g	31.17	b	41.37	c–f	28.27	a–c	1
26-10-06-03	33.50	c–g	29.08	f–l	44.10	bc	27.31	a–d	1
41-05-05-03	33.42	d–g	30.02	b–i	40.83	c–f	29.40	a	1
45-03-06-01	33.24	d–g	29.69	b–j	41.90	c–e	28.13	a–c	1
63-06-08-02	33.20	d–g	28.71	g–l	43.70	bc	27.20	a–e	1
46-06-04-01	33.18	d–g	31.04	b–d	39.83	d–g	28.65	a	1
Florunner	33.15	d–g	29.33	c–l	42.07	c–e	28.07	a–c	1
31-04-05-02	32.98	d–g	29.36	c–l	40.90	c–f	28.69	a	1
63-04-05-02	32.98	d–g	30.11	b–h	40.43	c–f	28.40	ab	1
31-04-10-03	32.94	d–g	29.27	d–l	41.60	c–f	27.94	a–c	1
60-02-02-03	32.92	d–g	29.44	b–k	40.77	c–f	28.57	ab	1
46-06-07-02	32.92	d–g	29.38	c–l	40.93	c–f	28.44	ab	1
44-10-04-02	32.92	d–g	30.01	b–i	41.37	c–f	27.38	a–c	1
63-04-02-03	32.91	d–g	29.80	b–i	40.67	c–f	28.25	a–c	1
50-02-04-01	32.79	d–g	28.30	i–l	42.10	c–e	27.97	a–c	1
41-10-03-03	32.78	d–g	28.38	h–l	41.83	c–e	28.13	a–c	1
43-09-02-03	32.72	d–g	28.45	h–l	41.60	c–f	28.10	a–c	1
43-09-03-01	32.39	e–g	27.69	kl	41.13	c–f	28.35	ab	1
63-04-02-02	32.31	e–g	30.80	b–f	41.63	c–f	24.50	ef	0
41-10-01-03	31.63	f–h	27.96	j–l	41.37	c–f	25.57	c–f	0
44-10-01-02	31.33	gh	29.20	e–l	38.93	e–g	25.85	b–f	0
70-03-07-01	29.78	h	27.87	kl	37.93	fg	23.53	f	0
19-05-06-03	29.65	h	27.81	kl	36.60	g	24.55	d–f	0
*p*	0.0003		0.0001		0.0001			0.0008	
Mean	33.15		29.58		42.06			27.79	
LSD	2.36		1.79		3.76			2.76	
CV	7.5%		3.7%		5.5%			5.8%	

^1^ N MFG, number of environments in which the accession occurred in the most favorable statistical group. ^2^ Values sharing the same letter are not statistically different at the *p* = 0.05 level of probability. ^3^ n.d., no data.

**Table 4:** Number of days for initial appearance of late leaf spot symptoms on 50% of plants.

Accession	Mean		Manga 2006		Nyankpala 2006		N MFG ^1^
63-04-05-02	52.12	a ^2^	51.33	a	52.91	a	2
63-04-02-02	51.67	ab	50.78	ab	52.55	ab	2
43-08-07-02	50.77	bc	49.71	a–c	51.83	ab	2
70-03-07-01	50.71	bc	49.38	a–d	52.03	ab	2
41-10-03-03	50.25	c	49.09	a–e	51.42	a–c	2
50-04-03-03	50.35	c	49.06	a–e	51.63	a–c	2
46-06-04-01	50.23	c	49.06	a–e	51.40	a–d	2
44-10-01-02	48.69	d	48.68	a–f	48.70	c–g	1
63-04-02-03	48.23	d	48.13	a–g	48.33	d–h	1
44-10-04-02	48.58	d	47.68	a–h	49.48	b–f	1
41-05-05-03	46.75	fg	46.88	b–h	46.61	e–j	0
46-06-07-02	48.09	de	46.50	b–h	49.69	b–e	0
31-04-05-02	46.77	fg	46.43	b–h	47.11	e–i	0
31-04-10-03	46.61	fg	46.35	c–i	46.86	e–j	0
43-09-03-01	46.90	ef	45.82	c–i	47.99	e–i	0
43-09-02-03	46.14	f–i	45.57	c–i	46.71	e–j	0
Florunner	45.89	f–j	45.07	d–i	46.71	e–j	0
41-10-01-03	46.41	f–h	45.05	d–i	47.77	e–i	0
26-10-06-03	46.21	f–i	45.02	d–i	47.40	e–i	0
45-03-06-01	45.65	f–k	44.96	e–i	46.33	g–j	0
50-04-01-03	45.58	g–k	44.82	e–j	46.33	g–j	0
60-02-02-03	45.12	i–k	44.66	f–j	45.58	h–j	0
45-04-02-01	45.32	h–k	44.64	f–j	46.00	g–j	0
63-06-08-02	45.54	g–k	44.63	f–j	46.44	f–j	0
43-08-06-02	45.87	f–j	44.63	f–j	47.11	e–i	0
55-437	44.85	jk	44.36	f–j	45.33	h–j	0
19-05-06-03	45.03	i–k	44.07	g–j	45.99	g–j	0
50-02-04-01	44.50	k	43.78	g–j	45.21	ij	0
43-09-03-02	44.66	jk	43.65	h–j	45.67	g–j	0
Chinese	42.99	l	41.99	ij	44.00	j	0
Spanish	42.23	l	40.45	j	44.00	j	0
*p*	0.0001		0.0029		0.0001		
Mean	46.07		46.20		47.91		
LSD	1.27		4.39		3.08		
CV	2.1%		4.7%		3.9%		

^1^ N MFG, number of environments in which the accession occurred in the most favorable statistical group. ^2^ Values sharing the same letter are not statistically different at the *p* = 0.05 level of probability.

**Table 5:** Pod yield (kg ha^−1^).

Accession	Mean		Manga 2006		Nyankpala 2006		Manga 2007		Nyankpala 2007		N MFG ^1^
43-09-03-01	2027	a ^2^	2400	a	336	a–c	3407	a	1966	a–c	4
44-10-01-02	1787	ab	2067	ab	342	a–c	2487	a–c	2251	ab	4
63-06-08-02	1546	a–c	933	c–g	314	a–c	2687	ab	2249	ab	3
43-09-02-03	1529	a–d	1733	a–c	294	b–e	2293	a–d	1793	b–d	2
43-08-06-02	1518	b–d	867	d–g	398	ab	2013	b–i	2795	a	2
43-09-03-02	1476	b–d	1170	c–f	270	c–h	2347	a–d	2103	a–c	2
46-06-07-02	1394	b–e	1600	a–d	261	c–i	2260	a–e	1453	b–f	2
19-05-06-03	1358	b–f	1533	b–e	286	b–f	2233	a–f	1627	b–e	1
41-10-01-03	1276	c–g	933	c–g	342	a–c	2033	b–h	1795	b–d	1
26-10-06-03	1262	c–g	833	d–g	227	c–i	1833	b–i	2153	a–c	1
45-04-02-01	1245	c–g	933	c–g	284	b–g	2173	a–g	1590	b–e	1
50-02-04-01	1224	c–h	1067	c–g	305	a–d	1553	b–i	1969	a–c	2
63-04-05-02	1219	c–h	933	c–g	171	e–j	1700	b–i	2073	a–c	1
44-10-04-02	1196	c–i	933	c–g	271	c–h	2280	a–e	1301	c–f	0
Florunner	1134	c–i	500	fg	228	c–i	2147	b–g	1661	b-e	0
41-10-03-03	1125	c–i	667	fg	259	c–i	1740	b–i	1835	b–d	0
43-08-07-02	1097	c–i	1267	b–f	285	b–f	1420	c–i	1417	b–f	0
Spanish	1026	d–j	1000	c–g	142	ij	1540	b–i	1421	b–f	0
31-04-05-02	954	e–k	933	c–g	252	c–i	1373	c–i	1257	c–f	0
45-03-06-01	939	e–k	867	d–g	138	ij	1760	b–i	991	d–f	0
50-04-03-03	913	e–k	733	e–g	217	c–i	1753	b–i	949	d–f	0
41-05-05-03	878	f–k	833	d–g	312	a–c	1113	d–i	1255	c–f	1
60-02-02-03	841	g–k	733	e–g	160	f–j	1000	f–i	1470	b–f	0
50-04-01-03	831	g–k	1333	b–f	168	f–j	1200	d–i	622	f	0
70-03-07-01	779	g–k	733	e–g	181	d–j	1213	d–i	989	d–f	0
46-06-04-01	721	h–k	667	fg	240	c–i	967	g–i	1009	d–f	0
63-04-02-03	712	i–k	567	fg	155	h–j	1047	e–i	861	ef	0
31-04-10-03	565	jk	667	fg	229	c–i	793	i	570	f	0
63-04-02-02	501	k	267	g	158	g–j	887	hi	625	f	0
Chinese	n.d.		733	e–g	425	a	n.d.		n.d.		0
55-437	n.d.		n.d.		55	j	1600	b–i	799	ef	0
*p*	<0.0001		0.0003		<0.0001		0.0161		0.0002		
Mean	1140		1006		249		1762		1495		
LSD	506		895		127		1239		916		
CV	54.0%		48.1%		31.2%		43.0%		35.4%		

^1^ N MFG, number of environments in which accession occurred in most favorable statistical group. ^2^ Values sharing the same letter are not statistically different at the *p* = 0.05 level of probability. ^3^ n.d., no data.

**Table 6:** Pearson correlations among traits, by site.

**2006 Manga**	**AUDPCELS**		**AUDPCLLS**		**T50ELS**		**T50LLS**		**PODYIELD**	
AUDPCELS	1.0000									
AUDPCLLS	0.8423	*** ^2^	1.0000							
T50ELS	n.d.		n.d.		n.d.					
T50LLS	−0.1654		−0.3492	**	n.d.		1.0000			
PODYIELD	0.2675	*	0.2768	**	n.d.		0.0913		1.0000	
**2006 Nyankpala**	**AUDPCELS**		**AUDPCLLS**		**T50ELS**		**T50LLS**		**PODYIELD**	
AUDPCELS	1.0000									
AUDPCLLS	0.7735	***	1.0000							
T50ELS	0.2565	*	0.2740	**	1.0000					
T50LLS	−0.1009		−0.3126	**	0.1037		1.0000			
PODYIELD	−0.1271		−0.0050		−0.2816	**	−0.0379		1.0000	
**2007 Manga**	**AUDPCELS**		**AUDPCLLS**		**T50ELS**		**T50LLS**		**PODYIELD**	
AUDPCELS	1.0000									
AUDPCLLS	0.6608	***	1.0000							
T50ELS	−0.0160		0.0409		1.0000					
T50LLS	n.d.		n.d.		n.d.		n.d.			
PODYIELD	−0.0613		−0.1501		0.1178		n.d.		1.0000	
**2007 Nyankpala**	**AUDPCELS**		**AUDPCLLS**		**T50ELS**		**T50LLS**		**PODYIELD**	
AUDPCELS	1.0000									
AUDPCLLS	0.8104	***	1.0000							
T50ELS	0.0483		−0.0492		1.0000					
T50LLS	n.d.		n.d.		n.d.		n.d.			
PODYIELD	−0.0634		−0.1161		0.0551		n.d.		1.0000	

^1^ AUDPCELS, AUDPC for early leaf spot; AUDPCLLS, AUDPC for late leaf spot; T50ELS, days to early leaf spot lesions on 50% of plants; T50LLS, days to appearance of late leaf spot; PODYIELD, pod yield per plot in kg ha^−1^. ^2^ *, significant at the *p* = 0.05 level of probability; **, significant at *p* = 0.01; *** significant at *p* = 0.001. ^3^ n.d., no data.

## Data Availability

Datasets including leaf spot scores and pod yields, time to 50% infection, and the CalcIP6 software source code and executable file used to estimate time to 50% infection have been placed online at Figshare (www.figshare.com). Files may be accessed at https://doi.org/10.6084/m9.figshare.14482791.

## Supplementary Materials

The following are available online at https://www.mdpi.com/article/10.3390/plants10050873/s1: Supplementary Table S1, Analysis of variance table; Supplementary Table S2, Summary of selections; Supplementary Table S3, CalcIP6.c—computer program to interpolate percentages of diseased plants over time, and estimate days to 50% infected plants.
