# Supplementary material for: Evaluation and Selection of Interspecific Lines of Groundnut (Arachis hypogaea L.) for Resistance to Leaf Spot Disease and for Yield Improvement
Source: Plants (Basel). 2021 Apr 26;10(5):873. doi: 10.3390/plants10050873 (PMC8146533; doi:10.3390/plants10050873)
Supplement: Supplementary file 1 [file plants-10-00873-s001.zip › sup/Supplementary_Table1_v2.pdf]

Supplementary Table 1. Analysis of variance for traits in Tables 1-5.

Trait: AUDPC for early leafspot for Table 1.

**Source**	**SS**	**df**	**MS**	**F**	**P**
Environment	2,056,400	3	685,468.3	361.04	<0.0001
Rep (Environment)	15,189	8	1,898.6	5.94	<0.0001
Genotype	37,139	29	1,280.7	2.10	0.0044
Environment x Genotype	53,085	87	610.2	1.91	0.0001
Error	71,009	222	319.9		

Trait: AUDPC for early leafspot for Table 2.

**Source**	**SS**	**df**	**MS**	**F**	**P**
Environment	1,351,970	3	450,658.3	266.26	<0.0001
Rep (Environment)	13,540	8	1,692.5	5.69	<0.0001
Genotype	29,286	29	1,009.9	1.43	0.1024
Environment x Genotype	61,254	87	704.1	2.37	<0.0001
Error	66,068	222	297.6		

Trait: Time to appearance of symptoms for early leafspot for Table 3.

**Source**	**SS**	**df**	**MS**	**F**	**P**
Environment	10,940	2	5,470.09	1169.10	<0.0001
Rep (Environment)	28	6	4.68	1.54	0.1667
Genotype	517	30	17.23	2.81	0.0003
Environment x Genotype	367	60	6.12	2.02	0.0002
Error	531	175	3.03		

Trait: Time to appearance of symptoms for late leafspot for Table 4.

**Source**	**SS**	**df**	**MS**	**F**	**P**
Environment	17.1	1	17.13	0.12	0.7493
Rep (Environment)	419.1	3	139.69	35.71	<0.0001
Genotype	921.9	30	30.73	31.94	<0.0001
Environment x Genotype	28.9	30	0.96	0.25	>0.9999
Error	352.0	90	3.91		

Trait: Pod yield for Table 5.

**Source**	**SS**	**df**	**MS**	**F**	**P**
Environment	116,617,000	3	38,872,400	17.21	0.0008
Rep (Environment)	18,070,100	8	2,258,770	8.30	<0.0001
Genotype	41,724,200	28	1,490,150	3.85	<0.0001
Environment x Genotype	32,515,100	84	387,085	1.42	0.0221
Error	59,598,700	219	272,140		
